# Using Wild Olives in Breeding Programs: Implications on Oil Quality Composition

**DOI:** 10.3389/fpls.2018.00232

**Published:** 2018-02-27

**Authors:** Lorenzo León, Raúl de la Rosa, Leonardo Velasco, Angjelina Belaj

**Affiliations:** ^1^IFAPA Centro Alameda del Obispo, Córdoba, Spain; ^2^Instituto de Agricultura Sostenible – Consejo Superior de Investigaciones Científicas, Córdoba, Spain

**Keywords:** breeding, fatty acid composition, minor components, *Olea europaea*, oleasters

## Abstract

A wide genetic diversity has been reported for wild olives, which could be particularly interesting for the introgression of some agronomic traits and resistance to biotic and abiotic stresses in breeding programs. However, the introgression of some beneficial wild traits may be paralleled by negative effects on some other important agronomic and quality traits. From the quality point of view, virgin olive oil (VOO) from olive cultivars is highly appreciated for its fatty acid composition (high monounsaturated oleic acid content) and the presence of several minor components. However, the composition of VOO from wild origin and its comparison with VOO from olive cultivars has been scarcely studied. In this work, the variability for fruit characters (fruit weight and oil content, OC), fatty acid composition, and minor quality components (squalene, sterols and tocopherols content and composition) was studied in a set of plant materials involving three different origins: wild genotypes (*n* = 32), cultivars (*n* = 62) and genotypes belonging to cultivar × wild progenies (*n* = 62). As expected, values for fruit size and OC in wild olives were lower than those obtained in cultivated materials, with intermediate values for cultivar × wild progenies. Wild olives showed a remarkably higher C16:0 percentage and tocopherol content in comparison to the cultivars. Contrarily, lower C18:1 percentage, squalene and sterol content were found in the wild genotypes, while no clear differences were found among the different plant materials regarding composition of the tocopherol and phytosterol fractions. Some common highly significant correlations among components of the same chemical family were found in all groups of plant materials. However, some other correlations were specific for one of the groups. The results of the study suggested that the use of wild germplasm in olive breeding programs will not have a negative impact on fatty acid composition, tocopherol content, and tocopherol and phytosterol profiles provided that selection for these compounds is conducted from early generations. Important traits such as tocopherol content could be even improved by using wild parents.

## Introduction

The use of novel genetic diversity in plant breeding is considered of paramount importance to obtain new cultivars adapted to high productive, resilient, and sustainable growing systems. Crop wild relatives represent potential new sources of genetic diversity so that global conservation priorities for crop wild genetic resources are encouraged (Castañeda-Álvarez et al., 2016). According to recent revisions, the species to which cultivated olive belongs, *Olea europaea*, includes six sub-species based on morphology and geographical distribution (Green, 2002). Among them, the subsp. *europaea*, which can be found throughout the whole Mediterranean basin, is represented by two botanical varieties: cultivated olive (var. *europaea*) and wild olive (var. *sylvestris*). A wide genetic diversity has been reported for wild olives, even higher than the observed for cultivated ones (Baldoni et al., 2006; Breton et al., 2006; Belaj et al., 2010; Erre et al., 2010; Boucheffa et al., 2016). This could be particularly interesting for the introgression of some agronomic characters in breeding programs, such as biotic and abiotic stresses resistance, as already reported for many crops (Hajjar and Hodgkin, 2007). For instance, resistance to verticillium wilt scarcely found in current cultivars, has been observed in wild germplasm (Colella et al., 2008; Arias-Calderón et al., 2015), which suggest its potential use as parents in olive breeding programs. Progenies involving wild parents have also showed shorter juvenile period and more abundant flowering than progenies from cultivated parents, which can represent important advantages for olive breeding (Klepo et al., 2013, 2014). However, it is not fully understood whether the introgression of some beneficial characters could be accompanied by a parallel negative effect regarding some other important agronomic and quality characters.

From the quality point of view, virgin olive oil (VOO) is highly appreciated due to its fatty acid (high monounsaturated oleic acid content) and minor compounds composition. All these components are responsible of well-known healthy properties of VOO (Pérez-Jiménez et al., 2007). High variability for most olive oil quality components has been reported in progenies from breeding programs (Sánchez de Medina et al., 2014, 2015; De la Rosa et al., 2016). However, compositional quality of VOOs from wild origin has been scarcely studied. Some works comparing oil composition between cultivars and wild olives indicate overlapping results between the two groups (Hannachi et al., 2008; Dabbou et al., 2011; Boucheffa et al., 2014). While it seems that fruit size and oil content (OC) are determinant traits to discriminate between wild and cultivated forms (Hannachi et al., 2008; Belaj et al., 2011). Wild olives yielding high quality oils were suggested to be commercially useful (Baccouri et al., 2008; Dabbou et al., 2011), although a possible feral origin of the genotypes reported to have high OC cannot be excluded (Baccouri et al., 2008).

Up to recently, most of the olive breeding programs were based in intra-specific crosses between cultivars of well-known merit (De la Rosa et al., 2013), representing the selection work performed in Australia a rare case of the use of wild olive material for olive breeding. As a result, based on oil yield and quality, some interesting and well adapted genotypes to the conditions of Australia were selected, although feral but not genuine wild olives were used (Sedgley, 2004). In this sense, in the framework of the olive breeding program of Córdoba Spain, many efforts have been dedicated, in the last decade, to the collection, *ex situ* conservation and evaluation of wild genotypes from different origins (Belaj et al., 2007, 2011) as well as initial characterization of some olive progenies involving wild genotypes as parents (Klepo et al., 2013, 2014).

In this work, the variability for oil quality components including fatty acid composition and minor components such as tocopherols, phytosterols, and squalene was studied in a set of plant materials involving wild genotypes, cultivars, and crosses between them. The main objective of the research was to study the usefulness and implications for oil quality of using wild genetic resources in olive breeding. Additionally, we intended to gain some insights about the genetic determinism for these characters.

## Materials and Methods

### Plant Materials

The olive germplasm under study include genotypes from three different origins: wild genotypes (*n* = 32), cultivars (*n* = 62) and genotypes belonging to cultivar × wild progenies (*n* = 62). All the genotypes were grown in the same conditions at the IFAPA Centro Alameda del Obispo, Córdoba (Spain). Maintained in an *ex situ* wild olive collection (De la Rosa et al., 2014), the wild genotypes came from prospecting surveys in Andalusia and Balearic Islands (21 and 9, respectively), and the rest (2) belonged to subs *Olea guanchica* from Canary Islands. The cultivated plant material under study comprised 62 olive cultivars from 13 different countries maintained at the World Olive Germplasm Collection (Supplementary Table 1). In both cases, each genotype is represented by two trees which have been evaluated for the traits under study during two harvesting seasons. In addition, 62 genotypes from crosses between the cultivar ‘Picual’ and two wild trees (W1 and W2), including 44 and 18 genotypes, respectively, were also evaluated. In this case, each seedling is represented by a single tree. Most of the plant materials were evaluated in two harvest seasons. However, average values per genotype were used in all analysis as genotype effect represented the main source of variation accounting for a major proportion of sums of squares for the evaluated traits, compared to harvest season effect (Supplementary Table 2).

### Traits Evaluated

Fruit samples of around 0.5 kg were randomly collected for each plant in a common date (mid-November), typical for olive harvesting. Previous works suggest that it seems more efficient to compare genotypes in a common date rather than to harvest the olive samples in a fixed ripening index, which is also quite difficult to achieve for large number of genotypes (De la Rosa et al., 2013; Bodoira et al., 2016). From each sample, three subsamples of around 25 g were randomly selected to produce dried samples sizes suitable for NMR sample holder. Fruit fresh weight (FFW) was measured and, after drying in a forced-air oven at 105°C for 42 h to ensure dehydration, OC was determined using an NMR fat analyzer (Minispec MQone, Bruker Optik GmbH, Ettlingen, Germany), and expressed as a percentage on dry weight basis.

Twenty additional fruits were randomly chosen from each sample, stored at -80°C and then lyophilized. After lyophilization, the stones were removed and the flesh was milled in a laboratory ball mill. The samples were then stored at -20°C till analysis, usually within 48–72 h. All the analyses were performed in duplicate following previous procedures developed in our breeding program for direct analysis of fruit flesh (Velasco et al., 2014). In short, fatty acid composition was analyzed by simultaneous oil extraction and fatty acid methylation followed by gas-liquid chromatography (GLC) on a Perkin Elmer Clarus 600 GC (Perkin Elmer Inc., Waltham, MA, United States) equipped with a BPX70 30 m × 0.25 mm internal diameter × 0.25 μm film thickness capillary column (SGE Analytical Science Pty Ltd., Ringwood, VIC, Australia). Fatty acids were named according to C:D nomenclature, number of carbon atoms:number of double bonds in the chain. Tocopherol extraction, separation by high-performance liquid chromatography (HPLC), and quantification was done on around 100 mg of lyophilized olive flesh using a fluorescence detector (Waters 474) at 295 nm excitation and 330 nm emission and iso-octane/tert-butylmethylether (94:6) as eluent at an isocratic flow rate of 0.8 ml/min. Quantitative determination of tocopherols was done by using rac-5,7-dimethyltocol (Matreya LLC, Pleasant Gap, PA, United States) as internal standard and total tocopherol content (TO) was calculated as the sum of α, β, γ and δ-tocopherol contents, expressed as mg kg^-1^ lyophilized fruit flesh. Finally, sterols (ST) and squalene (SQ) contents in lyophilized olive flesh samples were analyzed by GLC of the unsaponifiable fraction following silylation, using a Perkin Elmer Clarus 600 Gas Chromatograph equipped with a ZB-5 capillary column (id = 0.25 mm, length = 30 m, film thickness = 0.10 μm; Phenomenex, Torrance, CA, United States). Total sterol content was expressed as mg kg^-1^ lyophilized fruit flesh.

### Data Analysis

Principal components analysis (PCA) was used to investigate the variability between and within the different groups of samples evaluated and the relationship among traits, and analysis of variance and box and whiskers plots were performed for the most important variables selected from PCA to analyze differences between groups. Pearson correlation and linear regression were used to test the relations among the traits measured. Unscrambler (CAMO A/S, Trondheim, Norway) and Statistix (Analytical Software, Tallahassee, FL, United States) software were used for the statistical analysis.

## Results and Discussion

**FIGURE 1:**
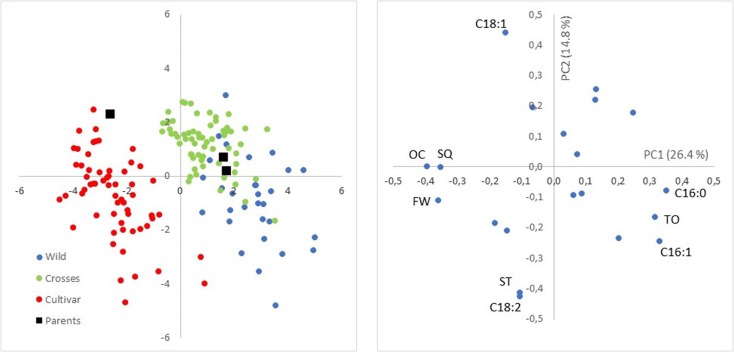
Score **(Left)** and loading **(Right)** plots of the first two components accounting for 26.4 and 14.8% of total variability. Wilds, cultivars, crosses, and parents of the two crosses are indicated in the score plot, while main traits contributing to loadings are identified in the loading plot.

PCA was used for a preliminary exploratory analysis of variability between and within groups of samples (**Figure 1**). The score biplot of PC1 (26.4% of total variation) and PC2 (14.8%) showed clear separation of the three groups of samples (wilds, cultivars, and crosses), although a wide variability was also observed within each of the three groups. PC1 was positively correlated mainly with C16:0, C16:1, and TC, and negatively with FFW, OC, and SQ content. PC2 was associated positively mainly with C18:1 and negatively with C18:2 and ST. Main separation between groups was obtained through PC1, i.e., higher values for C16:0, C16:1, and TC are expected for wilds, and higher values for FW, OC, and SQ content for cultivars, with intermediate values in progenies from crosses in accordance to the relative position of the parents in the score plot.

**FIGURE 2:**
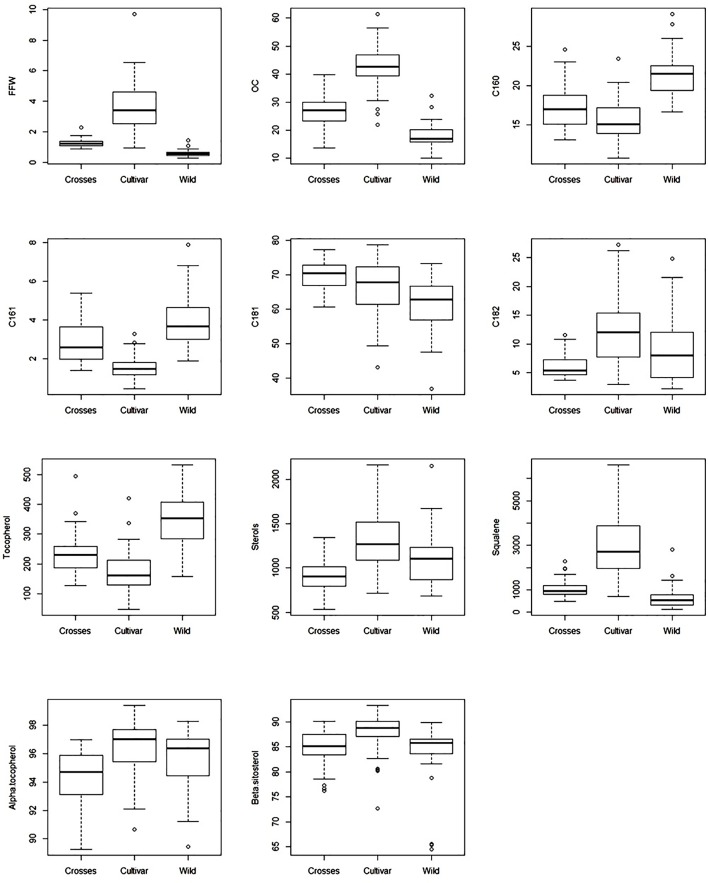
Box and whisker plots for fruit traits and main oil quality components evaluated in the three groups of samples (wilds, cultivars, and crosses).

The general results inferred from PCA could be expanded to original data, with significant differences in average values between groups of samples for the most important variables selected from PCA (**Table 1**). A wide and similar variability was observed in all three groups but clear differences in range of variation could be inferred from box and whiskers plots (**Figure 2**). High variability for most of the components has been reported in other cultivars and wilds collections (Aparicio and Luna, 2002; Baccouri et al., 2008; Hannachi et al., 2013; Beltrán et al., 2015; Kyçyk et al., 2016), as well as progenies from breeding programs (De la Rosa et al., 2016). From the commercial point of view, this wide variability could impose some restrictions according to international trade standards (International Olive Council [IOC], 2016) (**Table 2**). For instance, C16:0 values for most of the wild genotypes would be higher than established (20% maximum). Some of them could also affront problems due to currents limits for C18:1 and C18:2. It should be noted, however, that the same problem is currently faced for traditional cultivars with some of them exceeding thresholds values for the main fatty acids set up by the International Olive Council (International Olive Council [IOC], 2016). Interestingly, genotypes from crosses exceeded maximum permitted values for C16:0, probably due to the high content of wild parents, but they complied with the other thresholds. Similar results have been reported in previous evaluations. For instance, Baccouri et al. (2011) found that only 85 out of 150 studied wild olives showed an oil fatty acid composition within IOC trade standards.

**Table 1:** Descriptive statistics for fruit traits and main oil quality components evaluated in the three groups of samples (wilds, cultivars, and crosses).

Trait	Wild (*n* = 32)			Crosses (*n* = 62)			Cultivars (*n* = 62)		
	Mean		CV (%)	Mean		CV (%)	Mean		CV (%)
FFW (g)	0.60	c^∗^	42.33	1.24	b	19.68	3.66	a	44.39
OC (%)	18.03	c	25.72	26.74	b	20.83	42.57	a	17.49
C16:0 (%)	21.34	a	13.49	17.09	b	15.28	15.44	c	15.35
C16:1 (%)	3.91	a	36.42	2.83	b	38.28	1.55	c	37.92
C18:1 (%)	61.57	c	13.19	69.87	a	6.01	66.95	b	11.72
C18:2 (%)	9.03	b	60.86	6.06	c	33.50	12.40	a	46.91
Squalene (mg kg^-1^)	674.3	b	80.57	1048.8	b	35.05	3009.6	a	46.72
Tocopherol (mg kg^-1^)	347.1	a	28.61	233.4	b	25.34	172.0	c	38.90
α-tocopherol (%)	95.52	a	2.19	94.36	b	2.01	96.47	a	1.98
Sterols (mg kg^-1^)	1113.7	b	28.20	916.2	c	19.43	1310.2	a	25.58
β-sitosterol (%)	83.63		7.73	84.79		3.96	87.96		3.99

^∗^*For each trait different letters indicate significant differences between groups at *P* < 0.05*.

**Table 2:** Number of genotypes for each group exceeding thresholds values for the main fatty acids according to IOC trade standards (International Olive Council [IOC], 2016).

	C160		C181		C182	
	<7.5	>20.0	<55.0	>83.0	<2.5	>21.0
Wilds (32)	–	24	6	–	1	2
Cultivars (62)	–	3	5	–	–	5
Parents:						
Picual	13.93		77.33		3.52	
W1	17.64		69.08		6.13	
W2	21.39		62.25		6.30	
Crosses (62)	–	9	–	–	–	–

*Values of the parents for each fatty acid are indicated.*

As expected from PCA and previous results, wild olives were characterized by low average values of fruit size and OC, significantly lower than cultivated materials (Hannachi et al., 2008, 2009; Belaj et al., 2011), while intermediate ones were obtained in progenies from crosses. For all groups, C18:1 was clearly the most abundant fatty acid, followed by C16:0 and C18:2 (**Table 1**). Wilds showed the highest mean content for C16:0 and C16:1, cultivars the opposite results, and progenies from crosses intermediate values. Not so clear results were obtained for C18:1 and C18:2, where progenies from crosses showed the highest and lowest mean values, respectively. However, it should be noted that high oleic acid ‘Picual’ cultivar was used as parent for these crosses, which may have affected these results due to the high heritability reported for this character (De la Rosa et al., 2016). Similar average composition has been previously reported in the evaluation of wild olives from different origins, such as Tunisia (Baccouri et al., 2011), Turkey (Matthäus et al., 2014), and Pakistan (Anwar et al., 2013). In contrast with our results, much lower values of C16:0 were found in wilds olives from Algeria (Boucheffa et al., 2014) and Tunisia (Dabbou et al., 2011). Besides, no differentiation in the distribution of fatty acid composition was observed between wild and cultivated olives in Tunisia (Hannachi et al., 2008; Dabbou et al., 2011). In addition, extreme values have been reported for subsp. *cuspidata* specimens from Kenya, with lower C18:1 content (up to 44.3%) and subsequently higher C18:2 content (up to 33.3%) than those identified in the present research for wilds. Such contrasting results found in wild materials may be probably attributed to the different environmental factors linked to the geographical position where the genotypes were evaluated *in situ* (Hannachi et al., 2009), to the representativeness of the oleasters included in the studies (Dabbou et al., 2011) as well as to a possible feral origin of them (Hannachi et al., 2008; Dabbou et al., 2011).

Significant differences between groups were also obtained for total amounts of minor components, with cultivars showing the highest total values for SQ and ST and the lowest values for TO. Wilds showed the lowest average value for SQ and the highest average value for TO. High TO content has been previously reported in samples from wild olives (Baccouri et al., 2008; Boucheffa et al., 2014). The lowest average value for ST was found in the progenies from crosses. Again, low ST content of ‘Picual’ parent could have affected these results due to the high heritability also reported for this character (De la Rosa et al., 2016). No or only minimum differences were obtained regarding minor components composition, being α-tocopherol and β-sitosterol the predominant TO and ST forms, respectively, in all groups. The ST profile in wild olives is in agreement with the results obtained by Hannachi et al. (2013), although the authors did not quantify total ST content. To the best of our knowledge, no information on total SQ content in wild olive germplasm is found in the literature.

Pearson’s correlation coefficients among individual components reflected relationships previously inferred from PCA analysis (**Table 3**). Some highly significant values among components of the same chemical family were found in all groups of plant materials, for instance between α-tocopherol and γ-tocopherol or between δ5-avenasterol and β-sitosterol (data not showed), as previously reported for other materials in our breeding program (De la Rosa et al., 2016). However, no significant correlations were found among components of different chemical families.

**Table 3:** Correlations among fruit traits and main oil quality components evaluated in the three groups of samples: wilds, cultivars, and crosses from top to bottom.

	C16:0	C16:1	C18:1	C18:2	TO	SQ	ST
C16:0	x	0.72	-0.85	0.55	0.14	-0.18	0.03
	x	0.68	-0.81	0.60	0.34	-0.31	0.52
	x	0.90	-0.89	0.12	0.13	-0.31	0.06
C16:1	<0.0001	x	-0.48	0.12	0.02	-0.32	0.18
	<0.0001	x	-0.35	0.10	0.29	-0.24	0.43
	<0.0001	x	-0.81	0.09	0.24	-0.40	0.13
C18:1	<0.0001	0.0055	x	-0.90	0.02	0.24	0.04
	<0.0001	0.0048	x	-0.95	-0.29	0.41	-0.61
	<0.0001	<0.0001	x	-0.54	-0.13	0.40	-0.11
C18:2	0.0011	0.4986	<0.0001	x	-0.14	-0.20	-0.13
	<0.0001	0.4476	<0.0001	x	0.23	-0.42	0.54
	0.3656	0.4913	<0.0001	x	-0.05	-0.27	0.04
TO	0.4591	0.9109	0.9289	0.4326	x	0.21	0.32
	0.0064	0.0223	0.0206	0.0773	x	-0.49	0.23
	0.3211	0.0626	0.3009	0.6992	x	0.03	0.53
SQ	0.3108	0.0778	0.1882	0.2744	0.2444	x	-0.05
	0.0152	0.0650	0.0011	0.0007	<0.0001	x	-0.34
	0.0135	0.0012	0.0015	0.0327	0.8108	x	0.21
ST	0.8687	0.3144	0.8383	0.4843	0.0742	0.8012	x
	<0.0001	0.0006	<0.0001	<0.0001	0.0716	0.0066	x
	0.6523	0.3134	0.3818	0.7762	<0.0001	0.0977	x


*Pearson correlation coefficients and signification levels are indicated above and below the diagonal, respectively.*

**FIGURE 3:**
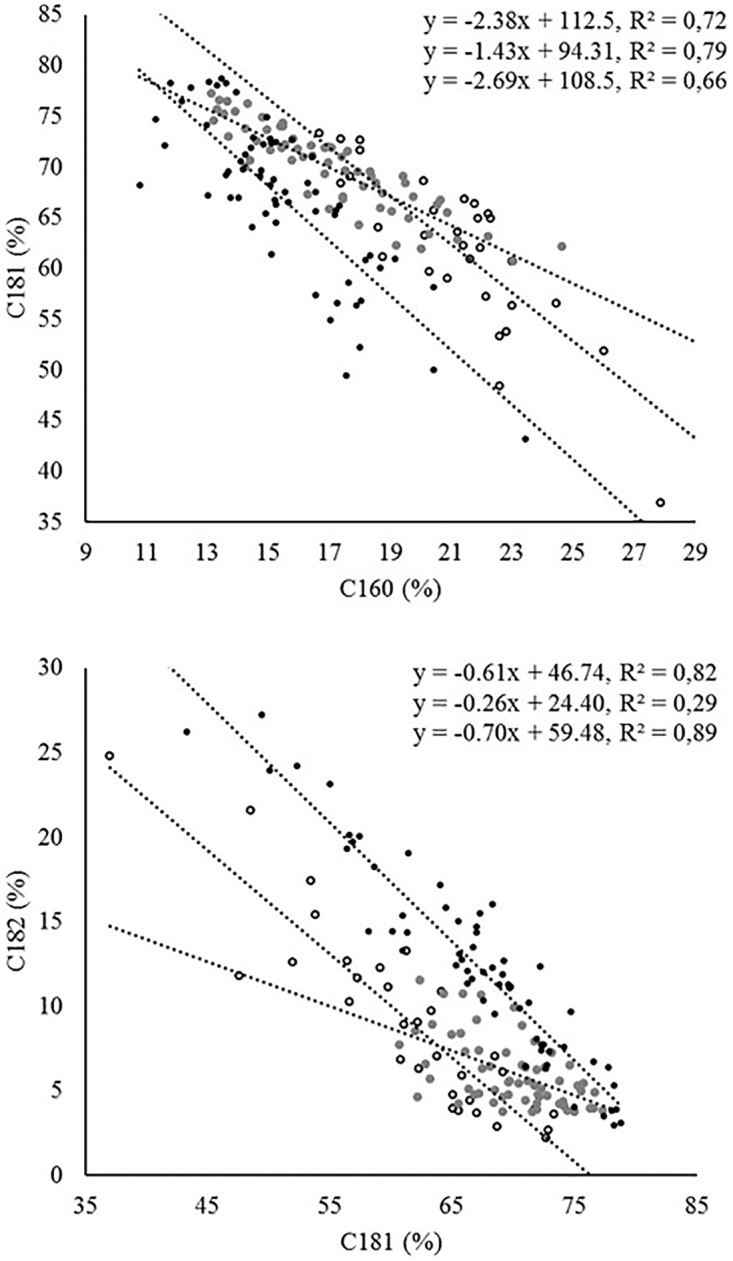
Linear regression between different fatty acid combinations in the three groups of samples: wilds (open circles), crosses (gray), and cultivars (black). Equations are presented for wilds, crosses, and cultivars from top to bottom, respectively.

In relation to the fatty acid profile, the most significant correlations were found between C16:0 and C16:1, which were positively correlated, and between C18:1 and both C16:0 and C18:2, in both cases negatively correlated. The evaluation of linear regressions between C18:1 and both C16:0 and C18:2 in the three groups of trees revealed marked differences in the coefficients of determination and/or the slope of the regression line (**Figure 3**). Thus, in the regression between C16:0 and C18:1, crosses showed a lower slope compared to cultivars and wilds. A similar situation was observed in the regression between C18:1 and C18:2 (**Figure 3**). In the latter case, the coefficient of determination was considerably lower in the crosses than in the parents and wilds, attributable to the existence of lower variability for both traits in the crosses than in the cultivated and wild parents (**Figure 3** and **Table 1**). The fatty acid composition of olive oil depends on both genetic and environmental factors, with genotypic variance having been reported as the main contributor to total variance in studies involving high genetic variability (León et al., 2008; Rondanini et al., 2011), as it is the case for the present study. The occurrence of strong negative correlation between oleic acid and both palmitic and linoleic acid has been reported previously in olive, which has been attributed to the relationship of these fatty acids in the biosynthetic pathway (Dabbou et al., 2012). Biosynthesis of C16 and C18 fatty acids in olive is located in plastids, where a series of enzymatic reactions that elongate an acyl chain bound to an acyl carrier protein (ACP) by the stepwise addition of two-carbon molecules are catalyzed by the fatty acid synthase complex. The last elongation step produces C18:0-ACP from C16:0-ACP. Then, C18:0-ACP can be desaturated by the C18:0-ACP desaturase to produce C18:1-ACP. The fatty acids C16:0, C18:0, and C18:1 can be exported from the plastid after the hydrolysis of the acyl-ACP by acyl-ACP thioesterases. Outside the plastid, C18:1 can be desaturated to C18:2 by the microsomal enzyme oleoyl-phosphatidylcholine desaturase (FAD2) (Somerville et al., 2000). This biosynthetic pathway is complex due to the existence of several rate-limiting reactions controlling carbon flux through the pathway, which determines that the levels of one fatty acid may alter the activity of other enzymes within the pathway and subsequently the levels of other fatty acids (Salas et al., 2014).

In summary, the results of the study seem to indicate that the use of wild germplasm in olive breeding programs could not have a negative impact on fatty acid composition, tocopherol content, and tocopherol and phytosterol profiles provided that selection for these compounds is conducted from early generations. Important traits such as TO content can be even improved by using wild parents. Conversely, our results indicated a putative negative impact on both total phytosterol and squalene contents, although this needs to be confirmed with studies involving larger populations.

## Author Contributions

All authors conceived and designed the experiment. AB was in charge of plant materials selection and samples collection. LL, RdlR, and LV performed fruit traits and oil quality analysis. LL prepared the first draft of the manuscript, all authors critically reviewed the manuscript prior to submission, read and approved the final version of the manuscript and agreed to be accountable for accuracy, integrity, and appropriateness of the manuscript.

## Conflict of Interest Statement

The authors declare that the research was conducted in the absence of any commercial or financial relationships that could be construed as a potential conflict of interest.
