# Supplementary material for: Using Wild Olives in Breeding Programs: Implications on Oil Quality Composition
Source: Front Plant Sci. 2018 Feb 27;9:232. doi: 10.3389/fpls.2018.00232 (PMC5835310; doi:10.3389/fpls.2018.00232)
Supplement: Supplementary file 1 [file Table_1.DOCX]

Supplementary Material

Using wild olives in breeding programs: implications on oil quality composition

Lorenzo León^1*^, Raúl de la Rosa^1^, Leonardo Velasco^2^, Angjelina Belaj^1^

*** Correspondence:** Corresponding Author: lorenzo.leon@juntadeandalucia.es

**Supplementary Table 1.-** Evaluated olive cultivars from the World Olive Germplasm Collection

| Cultivar | Origin |  | Cultivar | Origin |
| --- | --- | --- | --- | --- |
| Abou Chawkeh | Lebanon |  | Konservolia | Greece |
| Amygdalolia Nana | Greece |  | Koroneiki | Greece |
| Arbequina | Spain |  | Lastovka | Croatia |
| Ascolana Tenera | Italy |  | Leccino | Italy |
| Ayrouni | Lebanon |  | Lechin de Sevilla | Spain |
| Ayvalik | Turkey |  | Lucques | France |
| Azapa | Chile |  | Manzanilla Cacereña | Spain |
| Beladi o Souri | Lebanon |  | Manzanilla de Sevilla | Spain |
| Bianchera | Italy |  | Manzanilla Prieta | Spain |
| Blanqueta | Spain |  | Maurino | Italy |
| Bosana | Italy |  | Megaritiki | Greece |
| Bouteillan | France |  | Memecik | Turkey |
| Caninese | Italy |  | Meski | Tunisia |
| Carolea | Italy |  | Moraiolo | Italy |
| Changlot Real | Spain |  | Morisca | Spain |
| Chetoui | Tunisia |  | Oblica | Croatia |
| Cipresino | Italy |  | Pendolino | Italy |
| Cobrancosa | Portugal |  | Picholine | France |
| Coratina | Italy |  | Picholine Marocaine | MAR |
| Cordovil de Serpa | Portugal |  | Picual | Spain |
| Cornicabra | Spain |  | Picudo | Spain |
| Empeltre | Spain |  | Racimal | Spain |
| Fishomi | Iran |  | Rowghani | Iran |
| Franceantoio | Italy |  | Salonenque | France |
| Galega Vulgar | Portugal |  | Sevillenca | Spain |
| Gordal Sevillana | Spain |  | Toffahi | Egypt |
| Grossanne 67 | France |  | Uslu | Turkey |
| Hojiblanca | Spain |  | Valanolia | Greece |
| Istarska Bjelica | Croatia |  | Vera | Spain |
| Itrana | Italy |  | Verdale | France |
| Kalokerida | Greece |  |  |  |
