# Supplementary material for: Using Wild Olives in Breeding Programs: Implications on Oil Quality Composition
Source: Front Plant Sci. 2018 Feb 27;9:232. doi: 10.3389/fpls.2018.00232 (PMC5835310; doi:10.3389/fpls.2018.00232)
Supplement: Supplementary file 2 [file Table_2.DOCX]

Supplementary Material

Using wild olives in breeding programs: implications on oil quality composition

Lorenzo León^1*^, Raúl de la Rosa^1^, Leonardo Velasco^2^, Angjelina Belaj^1^

*** Correspondence:** Corresponding Author: lorenzo.leon@juntadeandalucia.es

**Supplementary Table 2.**- Percentage of sums of squares accounted by the different sources of variation for some of the evaluated traits.

| Source of variation | C18:1 (%) | Squalene (mg kg-1) | Tocopherol (mg kg-1) | Sterols (mg kg-1) |
| --- | --- | --- | --- | --- |
| Genotype | 93.8 | 82.0 | 71.3 | 77.6 |
| Year | 0.3 | 6.3 | 3.3 | 2.0 |
| Error | 5.9 | 11.7 | 25.4 | 20.3 |
